# New Way to Substitute Tetracyanocyclopropanes: One-Pot Cascade Assembling of Carbonyls and Malononitrile by the Only Bromine Direct Action

**DOI:** 10.5402/2011/469453

**Published:** 2011-07-26

**Authors:** Anatolii N. Vereshchagin, Michail N. Elinson, Nikita O. Stepanov, Gennady I. Nikishin

**Affiliations:** Homolytic Reactions Research Laboratory, N. D. Zelinsky Institute of Organic Chemistry, Leninsky prospect 47, 119991 Moscow, Russia

## Abstract

The new type of the chemical cascade reaction was found: formation of cyclopropanes from carbonyl compounds and CH acid by the only bromine direct action. The action of aqueous bromine on the carbonyl compounds and malononitrile in EtOH-H_2_O solutions in the presence of NaOAc results in the formation of 3-substituted 1,1,2,2-tetracyanocyclopropanes in 48–93% yields. The latter are well-known precursors for the different bicyclic heterosystems, among them those containing cyclopropane ring and those possessing different types of pharmacological activity.

## 1. Introduction

The cyclopropyl group is a vital structural unit in many synthetic and naturally occurring compounds, exhibiting a wide spectrum of biologic properties ranging from enzyme inhibition to herbicidal, antibiotic, antitumor, and antiviral activities [1–5]. Thus, the prevalence of cyclopropane containing compounds with biological activity, whether isolated from natural sources or rationally designed pharmaceutical agents, has inspired chemists to find novel and diverse approaches to their synthesis.

Though the methods of cyclopropanes synthesis have long been documented, so far, all of them consist of two main groups: (1) intramolecular cyclization or (2) interaction of two different molecules (addition of carbenes to olefins or Michael initiated ring closure (MIRC) are the most known examples of this type) [1, 3, 5].

**Figure 1:**
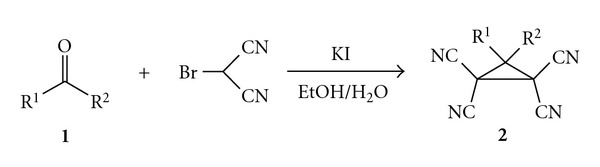
Wideqvist's condensation route to tetracyanocyclopropanes.

Nevertheless there are some special famous methods of the cyclopropane ring construction. One of them is well-known Wideqvist reaction, namely, the interaction of two molecules of bromomalononitrile with carbonyl compounds **1** in the presence of stoichiometric quantity of potassium iodide with the formation of the corresponding substituted tetracyanocyclopropanes **2** (Figure 1) [6].

Later in the electrochemical variant of Wideqvist reaction bromomalononitrile was replaced by malononitrile and catalytic amounts of sodium bromide [7, 8]. In the electrochemical variant for the reaction of aldehydes the low temperature 0°C is necessary [8], whereas for ketones a three- to fourfold excess of ketone is needed to obtain tetracyanocyclopropanes **2** in good yields [7, 8].

Recently we suggested a new strategy of the chemical route to the cyclopropane structure: the direct transformation of carbonyl compounds and malononitrile into 1,1,2,2-tetracyanocyclopropanes **2** [9]. Elemental bromine was used as active halogen compound, 1.2 equivalents of EtONa as base, and ethanol as solvent [9]. The next step of our research was direct one-pot transformation of alkylidenemalononitriles and malononitrile into substituted 1,1,2,2-tetracyanocyclopropanes **3** by the action of only bromine (without any base) in EtOH/H_2_O solution [10].

## 2. Results and Discussion

Cascade reactions have been utilized as powerful method to construct molecular complexity from readily available starting materials by combining two or more reactions into single transformation [11–13]. As such cascade reactions are of increasing importance in the modern organic chemistry. This is not only due to the need for the more efficient and less labour intense methodologies for the synthesis of organic compounds, but also consequence of the increasing importance of the environmental considerations in chemistry. Thus, cascade reactions have significant economical and ecological benefits when one performs several synthetic steps in one operation without isolating the reaction intermediates.

**Figure 2:**
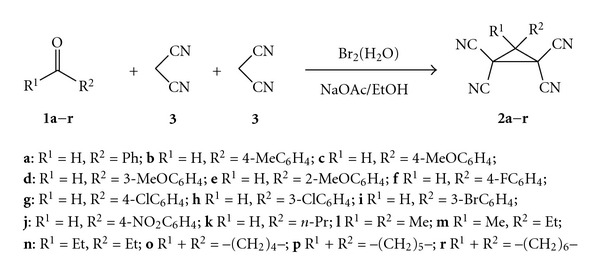
Direct one-pot cascade assembling of carbonyls and malononitrile into substituted 1,1,2,2-tetracyanocyclopropanes by the action of bromine in EtOH/H2O system.

Continuing our studies on functionally substituted cyclopropanes, we have found out that the action of aqueous bromine on the carbonyl compounds **1** and malononitrile **3** in aqueous ethanolic solution leads to 1,1,2,2-tetracyanocyclopropanes **2** (Figure 2).

First, to evaluate the synthetic potential of the procedure proposed and to optimize the general conditions, the cascade transformation of benzaldehyde **1a **with malononitrile, butanal **1k** with malononitrile and cyclohexanone **1p** with malononitrile into the substituted 1,1,2,2-tetracyanocyclopropanes **2a**, **2k**, and **2p**, respectively (Table 1).

**Figure 3:**
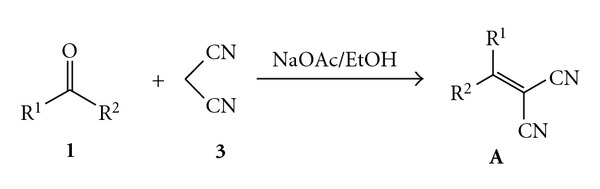
Knoevenagel condensation of the carbonyl compound and malononitrile.

The main idea of further experiments was to decrease the reactivity of elemental bromine to ensure the more selective cascade process of 3-substituted 1,1,2,2-tetracyanocyclopropane **2** synthesis. Thus, bromine was added not as elemental bromine but as 0.2 M solution in water. NaOAc acts as a catalyst for the Knoevenagel condensation of the carbonyl compound and malononitrile into alkylidenemalononitrile **A** (Figure 3).

In the case of alicyclic aldehydes and ketones NaOAc plays key role. Its presence increase the yields of products from 21% to 86% for 3-ethyl 1,1,2,2-tetracyanocyclopropane **2k** and from 43% to 86% for 1,1,2,2-tetracyanospiro[2.5]octane **2p**. Earlier NaOAc was used as a catalyst in the Perkin condensation [14] and in the Knoevenagel reaction [15].

Under the optimal conditions thus found carbonyl compounds **1a**–**r** and malononitrile **3** were transformed into corresponding substituted 1,1,2,2-tetracyanocyclopropanes **2a**–**r** in 43–93% yields (Table 2).

**Figure 4:**
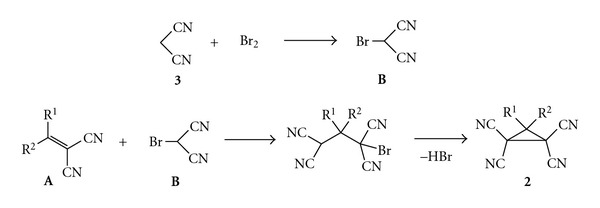
Mechanism of formation of substituted 1,1,2,2-tetracyanocyclopropanes **2**.

This process is typical cascade reaction which combines in the one-pot process four reactions, that is, (1) Knoevenagel condensation of the carbonyl compound and malononitrile into alkylidenemalononitrile **A** (Figure 3); (2) bromination of malononitrile, (3) Michael addition of bromomalononitrile **B** to alkylidenemalononitrile **A**, and (4) cyclization of substituted 1-bromo-1,1,3,3-tetracyanocyclo-propane into corresponding substituted 1,1,2,2-tetracyanocyclopropane **2** (Figure 4).

## 3. Conclusions

Thus, the new cascade reaction was found, namely, the direct formation of cyclopropane structures from carbonyl compounds and malononitrile by the direct action of the only bromine. The action of aqueous bromine on the equal amounts of carbonyl compounds and two equivalents of malononitrile in the presence of NaOAc in EtOH-H_2_O solution results in the formation of 3-substituted 1,1,2,2-tetracyanocyclopropanes in 43–93% yields. The latter are well-known precursors for the different bicyclic heterosystems, among them those containing cyclopropane ring [8, 16, 17] and those possessing different types of pharmacological activity [1–3, 18, 19]. The procedure utilizes inexpensive reagents; it is easily carried out and the work up is not complicated. 3-Substituted 1,1,2,2-tetracyanocyclopropanes are crystallized directly from the reaction mixture, consequently, the isolation includes only filtration.

## 4. Experimental Section

Chemicals were purchased from Aldrich and Acros. All melting points were measured with a Gallenkamp melting point apparatus and are uncorrected. ^1^H and ^13^C NMR spectra were recorded with a Bruker WM-250, Bruker AM-300 and Bruker Avance II 300 spectrometers at ambient temperature. Chemical shifts (*δ*) are given in ppm relative to Me_4_Si for [D_6_]DMSO and CDCl_3_ solutions. IR spectra were registered with a SPECORD M82 spectrometer in KBr pellets. Mass spectra (EI, 70 eV) were obtained directly using Finningan MAT INCOS 50 spectrometer.

### 4.1. General Procedure

To a mixture of carbonyl compound **1** (10 mmol), malononitrile (20 mmol), and sodium acetate (3 mmol) in 20 mL of ethanol in two necked 100 mL flask, 50 mL of 0.2 M bromine in water (10 mmol) was added dropwise during in 3 min. The mixture was magnetically stirred at 40°C for 1 h. Then solid phase was filtered off and dried to isolate pure tetracyanocyclopropane **2a**–**r**.

**3-Phenyl-1,1,2,2-tetracyanocyclopropane (2a)**

White solid. Yield 1.81 g (92%); m.p. 229–230°C (lit. m.p. [20] 227–230°C); ^1^H NMR (250 MHz, [D_6_]DMSO) 5.27 (s, 1 H, CH), 7.44–7.52 (m, 3 H, Ar), 7.74–7.82 (m, 2 H, Ar).

**3-(4-Methylphenyl)-1,1,2,2-Tetracyanocyclopropane (2b)**

White solid. Yield 1.67 g (91%); m.p. 226–229°C (lit. m.p. [20] 227–230°C); ^1^H NMR (300 MHz, [D_6_]DMSO) 2.33 (s, 3 H, CH_3_), 5.23 (s, 1 H, CH), 7.30 (d, *J* = 7.8 Hz, 2 H, Ar), 7.68 (d, *J* = 7.8 Hz, 2 H, Ar).

**3-(4-Methoxyphenyl)-1,1,2,2-Tetracyanocyclopropane (2c)**

Yellowish solid. Yield 1.91 g (93%); m.p. 208–210°C (lit. m.p. [20] 209–210°C); ^1^H NMR (300 MHz, [D_6_]DMSO) 3.79 (s, 3 H, OCH_3_), 5.15 (s, 1 H, CH), 7.04 (d, *J* = 8.4 Hz, 2 H, Ar), 7.73 (d, *J* = 8.4 Hz, 2 H, Ar).

**3-(3-Methoxyphenyl)-1,1,2,2-Tetracyanocyclopropane (2d)**

White solid. Yield 2.28 g (91%). m.p. 226–228°C (lit. m.p. [8] 227–230°C; ^1^H NMR (300 MHz, DMSO-d_6_) 3.76 (3 H, s, OCH_3_), 5.28 (1 H, s, CH), 7.15–7.45 (4 H, m, Ar).

**3-(2-Methoxyphenyl)-1,1,2,2-Tetracyanocyclopropane (2e)**

Yellowish solid. Yield 1.56 g (92%); m.p. 240–241°C (lit. m.p. [21] 240–241°C); ^1^H NMR (250 MHz, [D_6_]DMSO) 3.89 (s, 3 H, OCH_3_), 5.04 (s, 1 H, CH), 7.05 (t, *J* = 7.6 Hz, 1 H, Ar), 7.19 (d, *J* = 8.6 Hz, 1 H, Ar), 7.48 (t, *J* = 7.6 Hz, 1 H, Ar), 7.76 (d, *J* = 7.3 Hz, 1 H, Ar).

**3-(4-Fluorophenyl)-1,1,2,2-Tetracyanocyclopropane (2f)**

White solid. Yield 1.77 g (90%); m.p. 216–217°C (lit. m.p. [21] 216-217°C); ^1^H NMR (300 MHz, [D_6_]DMSO) 5.25 (s, 1 H, CH), 7.34 (t, *J* = 8.5 Hz, 2 H, Ar), 7.86–8.00 (m, 2 H, Ar).

**3-(4-Chlorophenyl)-1,1,2,2-Tetracyanocyclopropane (2g)**

White solid. Yield 2.07 g (85%); m.p. 250–251°C (lit. m.p. [20] 248–250°C); ^1^H NMR (300 MHz, [D_6_]DMSO) 5.28 (s, 1 H, CH), 7.59 (d, *J* = 8.5 Hz, 2 H, Ar), 7.88 (d, *J* = 8.5 Hz, 2 H, Ar).

**3-(3-Chlorophenyl)-1,1,2,2-Tetracyanocyclopropane (2h)**

White solid. Yield 1.65 g (87%); m.p. 187–189°C (lit. m.p. [20] 183–185°C); ^1^H NMR *δ*_H_ (300 MHz, [D_6_]DMSO) 5.35 (s, 1 H, CH), 7.51–7.59 (m, 2 H, Ar), 7.77–7.85 (m, 1 H, Ar), 8.08 (s, 1 H, Ar).

**3-(3-Bromophenyl)-1,1,2,2-Tetracyanocyclopropane (2i)**

White solid. Yield 1.81 g (92%); m.p. 186–187°C (lit. m.p. [21] 186–187°C); ^1^H NMR (250 MHz, [D_6_]DMSO) 5.32 (s, 1 H, CH), 7.45 (t, *J* = 8.5 Hz, 1 H, Ar), 7.69 (d, *J* = 8.5 Hz, 1 H, Ar), 7.88 (d, *J* = 8.5 Hz, 1 H, Ar), 8.23 (s, 1 H, Ar).

**3-(4-Nitrophenyl)-1,1,2,2-Tetracyanocyclopropane (3j)**

White solid. Yield 1.63 g (88%); m.p. 232–234°C (lit. m.p. [20] 232–235°C); ^1^H NMR (300 MHz, [D_6_]DMSO) 5.50 (s, 1 H, CH), 8.20 (d, *J* = 8.8 Hz, 2 H, Ar), 8.36 (d, *J* = 8.8 Hz, 2 H, Ar).

**3-n-Propyl-1,1,2,2-Tetracyanocyclopropane (2k)**

White solid. Yield 1.34 g (86%); m.p. 136–138°C (lit. m.p. [8] 136–138°C); ^1^H NMR (250 MHz, [D_6_]DMSO) 0.96 (t, *J* = 7.3 Hz, 3 H, CH_3_), 1.51–1.65 (m, 2 H, CH_2_), 1.69–1.77 (m, 2 H, CH_2_) 3.87 (t, *J* = 7.3 Hz, 1 H, CH).

**3,3-Dimethyl-1,1,2,2-Tetracyanocyclopropane (2l)**

White solid. Yield 0.73 g (55%); m.p. 208–210°C (lit. m.p. [22] 209.5–210°C); ^1^H NMR (300 MHz, [D_6_]DMSO): 1.58 (s, 6 H, CH_3_).

**3-Ethyl-3-Methyl-1,1,2,2-Tetracyanocyclopropane (2m)**

White solid. Yield 0.61 g (52%); m.p. 207–208°C (lit. m.p. [8] 208–209°C); ^1^H NMR (300 MHz, [D_6_]DMSO) 1.33 (t, *J* = 7.4 Hz, 3 H, CH_3_), 1.75 (s, 3 H, CH_3_), 2.16 (q, *J* = 7.4 Hz, 2 H, CH_2_).

**3,3-Diethyl-1,1,2,2-Tetracyanocyclopropane (2n)**

White solid. Yield 0.30 g (48%); m.p. 165–166°C (lit. m.p. [23] 167–168°C); ^1^H NMR (300 MHz, [D_6_]DMSO) 1.29 (t, *J* = 7.4 Hz, 6 H, CH_3_), 2.05 (q, *J* = 7.4 Hz, 4 H, CH_2_).

**1,1,2,2-Tetracyanospiro[2.4]heptane (2o)**

White solid. Yield 1.02 g (69%); m.p. 250–251°C (lit. m.p. [8] 250–251°C); ^1^H NMR (300 MHz, [D_6_]DMSO) 1.86–1.49 (m, 4 H, CH_2_), 2.01–2.07 (m, 4 H, CH_2_).

**1,1,2,2-Tetracyanospiro[2.5]octane (2p)**

White solid. Yield 1.57 g (75%); m.p. 178–180°C (lit. m.p. [22] 177–179°C); ^1^H NMR (300 MHz, [D_6_]DMSO) 1.46–1.56 (m, 2 H, CH_2_), 1.61–1.73 (m, 4 H, CH_2_), 1.80–1.90 (m, 4 H, CH_2_).

**1,1,2,2-Tetracyanospiro[2.6]nonane (2r)**

White solid. Yield 1.41 g (63%); m.p. 169–170°C (lit. m.p. [21] 170–171°C); ^1^H NMR (300 MHz, CDCl_3_) 1.70–1.79 (m, 4 H, CH_2_), 1.80–1.92 (m, 4 H, CH_2_), 2.08–2.16 (m, 4 H, CH_2_).

## Figures and Tables

**Table 1:** Direct transformation of benzaldehyde **1a**, butanal **1k** and cyclohexanone **1p** with malononitrile **3** into substituted 1,1,2,2-tetracyanocyclopropanes **2** ^a^.

Carbonyl	Solvent	Bromine	Catalyst	Yield of 2^b^ (%)
**1a**	EtOH	elemental	—	**2a**, 12
**1a**	EtOH/H_2_O	0.2 M (H_2_O)	—	**2a**, 90
**1a**	EtOH/H_2_O	0.2 M (H_2_O)	NaOAc	**2a**, 92
**1k**	EtOH/H_2_O	0.2 M (H_2_O)	—	**2k**, 21
**1k**	EtOH/H_2_O	0.2 M (H_2_O)	NaOAc	**2k**, 86
**1p**	EtOH/H_2_O	0.2 M (H_2_O)	—	**2p**, 43
**1p**	EtOH/H_2_O	0.2 M (H_2_O)	NaOAc	**2p**, 79

^a^10 mmol of carbonyl compound **1**, 20 mmol of malononitrile **3**, 5 mmol of NaOAc, 20 mL of EtOH, 50 mL of 0.2 M Br_2_ in water (10 mmol), temperature 40°C, time of reaction 1 hour.

^b^Yield of isolated product.

**Table 2:** Direct transformation of carbonyl compounds **1a**–**r** and malononitrile **3** into substituted 1,1,2,2-tetracyanocyclopropanes **2a**–**r** by the action of bromine in EtOH/water system^a^.

Olefin	R^1^	R^2^	Product	Yield of 2^b^ (%)
**1a**	H	Ph	**2a**	92
**1b**	H	4-MeC_6_H_4_	**2b**	91
**1c**	H	4-MeOC_6_H_4_	**2c**	93
**1d**	H	3-MeOC_6_H_4_	**2d**	91
**1e**	H	2-MeOC_6_H_4_	**2e**	92
**1f**	H	4-FC_6_H_4_	**2f**	90
**1g**	H	4-ClC_6_H_4_	**2g**	85
**1h**	H	3-ClC_6_H_4_	**2h**	87
**1i**	H	3-BrC_6_H_4_	**2i**	92
**1j**	H	4-NO_2_C_6_H_4_	**2j**	88
**1k**	H	n-Pr	**2k**	86
**1l**	Me	Me	**2l**	55
**1m**	Me	Et	**2m**	52
**1n**	Et	Et	**2n**	48
**1o**	– (CH_2_)_4_–		**2o**	69
**1p**	– (CH_2_)_5_–		**2p**	75
**1r**	– (CH_2_)_6_–		**2r**	67

^a^10 mmol of carbonyl compound **1**, 20 mmol of malononitrile **3**, 3 mmol of NaOAc, 20 mL of EtOH, 50 mL of 0.2 M Br_2_ in water (10 mmol), temperature 40°C, time of reaction 1 hour.

^b^Yield of isolated product.
